# The association between mental health, chronic disease and sleep duration in Koreans: a cross-sectional study

**DOI:** 10.1186/s12889-015-2542-3

**Published:** 2015-12-01

**Authors:** Min-Su Lee, Joon-Shik Shin, Jinho Lee, Yoon Jae Lee, Me-riong Kim, Ki Byung Park, Dongjin Shin, Jae-Heung Cho, In-Hyuk Ha

**Affiliations:** Jaseng Spine and Joint Research Institute, Jaseng Medical Foundation, 858 Eonju-ro, Gangnam-gu Seoul, 135-896 Republic of Korea; Department of Natural Science, Michigan State University, East Lansing, MI USA; Department of Korean Rehabilitation Medicine, College of Korean Medicine, Kyung Hee University, Seoul, Republic of Korea

**Keywords:** Sleep, Chronic disease, Mental disorders, Health surveys, Cross-sectional studies

## Abstract

**Background:**

Sleep duration holds considerable importance as an indicator of mental/physical health. The objective of this study was to investigate the association between sleep duration, mental health, and chronic disease prevalence in Koreans.

**Methods:**

Of 31,596 subjects eligible for the Korean National Health and Nutrition Examination Survey V (2010–2012), 17,638 participants who answered items on sleep duration (aged ≥19 yrs) were analyzed in a cross-sectional study. Association between sleep duration, mental health, and chronic disease prevalence was assessed using logistic regression, and adjusted for various socioeconomic and lifestyle characteristics.

**Results:**

Short or long sleep duration showed correlations with mental health, and items of significance showed gender-specific patterns. Women displayed significant associations with stress and depressive symptoms, and men with stress, thoughts of suicide, and psychiatric counseling. While stress was related with short sleep duration in both genders, depressive symptoms showed a relationship with long duration in men, and short duration in women. Prevalence of any chronic disease was associated with ≤6 h sleep when adjusted for factors including mental health, and among chronic diseases, cancer and osteoarthritis showed associations with short sleep duration, while diabetes and dyslipidemia were associated with normal sleep duration.

**Conclusions:**

Mental health problems were associated with sleep duration with gender-specific patterns. Associations with osteoarthritis, cancer, diabetes, dyslipidemia and abnormal sleep duration persisted after adjustment for mental health.

**Electronic supplementary material:**

The online version of this article (doi:10.1186/s12889-015-2542-3) contains supplementary material, which is available to authorized users.

## Background

Sleep is a daily process of restoration and recovery. Lack of sleep and sleep-related disorders are related with mental and physiological conditions, and may modulate the endocrine and immune systems and metabolism, consequently affecting quality of life, and emotional state. They may also increase mortality and risk of disease [1–3]. Despite individual differences, 7–8 h of sleep per day is generally considered appropriate to sustain physiological and mental health [4, 5].

Prior studies report that sleep duration is associated with various mental health aspects such as stress, depression, thoughts of suicide, mental disorders, and with chronic disease [6–12]. However, few studies have assessed the influence of different subcategories of mental health in men and women. This study investigated gender-specific patterns in associations between sleep duration and mental health.

Most previous studies failed to consider mental health in assessing the relationship between sleep duration and chronic disease, and it is uncertain whether sleep duration is affected by mental health factors consequent of chronic disease or by pathological expression of chronic disease itself. Despite results from a Brazilian population-based cross-sectional study on chronic disease and sleep that show depression and anxiety are related with sleep duration, it is limited in that it did not take into account whether depression and anxiety affected chronic diseases in the association between chronic disease and sleep duration [13]. A large-scale cohort by Ayas et al. reported that short and long sleep duration were both related to cardiovascular disease, but only depression was included as a covariate [14].

This study investigated whether sleep duration, mental health disorder and chronic disease prevalence were associated, and whether sleep duration displays gender-specific patterns in these associations using cross-sectional data from the Korean National Health and Nutrition Examination Survey (KNHANES).

## Methods

### Study population and sampling

This study was conducted using data obtained from the KNHANES V, which employed a rolling sampling method involving stratified, multistage, probability-clusters to yield a representative sample of South Koreans. The survey was administered by the Korean Ministry of Health and Welfare and was made up of 3 parts: health survey, nutrition survey, and health examination. Further details are listed in “The 5^th^ KNHANES Sample Design” and reports, made accessible on the KNHANES website https://knhanes.cdc.go.kr/knhanes/index.do. The KNHANES annual reports, user manuals and instructions, and raw data are available on request. The KNHANES V health examination and survey were completed by 24,173 participants (76.5 % of the target population, *n* = 31,596). Analysis was performed in 17,638 subjects aged ≥19 yrs who answered to survey items on sleep duration, and subjects with missing values in mental health, chronic disease, and covariates were excluded from each corresponding analysis.

### Sleep duration

Sleep duration was categorized as average ≤6 h, 7–8 h, and ≥9 h of sleep per day as classified in the health survey.

### Mental health

Stress perception was classified into high (very high or high degree of stress), low (low degree of stress), and almost none (almost no stress). Assessment of depressive symptom morbidity, psychiatric counseling, and thoughts of suicide was binary in the health survey: those with and those without experience of continuous depressive symptoms for ≥2 weeks; those who had and those who had not sought counseling for psychiatric issues during the past year; and those with and those without thoughts of suicide over the past year, respectively. Those who had contemplated suicide were further divided into those who had attempted suicide and those who had not.

The questions on sleep duration and mental health used in the survey are as follows:

- How many hours do you sleep a day? □□ hours
- How much stress do you feel in your usual daily activities?

① Very high stress levels
② High stress levels
③ Low stress levels
④ Almost no stress

- Have you experienced sadness or despair severe enough to interfere with daily activities for 2 consecutive weeks or longer during the previous year?

① Yes
② No

- Have you contemplated suicide over the previous year?

① Yes
② No

- Have you received psychiatric counseling through visits, by phone or internet during the previous year?

① Yes
② No

### Chronic disease

Chronic disease included hypertension, dyslipidemia, stroke, cardiovascular disease, rheumatoid arthritis, diabetes, atopic dermatitis, pulmonary tuberculosis, asthma, thyroid-related disorders, cancer, or hepatitis, and morbidity of any chronic disease and was assessed through self-reported survey items. Chronic disease morbidity was defined as morbidity for ≥3 months over the past year. Cardiovascular disease included myocardial infarction and angina; cancer included gastric cancer, liver cancer, colorectal cancer, breast cancer, cervical cancer, lung cancer, and other cancers; and hepatitis included hepatitis type B and C.

### Covariates

The general, socioeconomic, lifestyle-related, and health-related characteristics (age, sex, income, education, occupation, marital status, drinking, smoking, subjective health, BMI, use of medical care, level of daily activity, bed rest for the past month, absence from work for the past month) were assessed. Gender considered men and women, and age evaluated as continuous variables. Education was categorized into 4 levels: ≤8, 9–11, 12–15, and ≥16 years of formal education. Income level was evaluated as quartile ranges by monthly average household income with equalization (monthly household income divided by number of household members). Employment status was assessed as employment and unemployment. Smoking was divided into never smokers, exsmokers, and current smokers, and drinking habits into 4 levels: (a) non alcohol consumption with no alcohol consumption for the past year; (b) <1 drinking episode per month; (c) <5 drinking episodes per month (1–4 episodes per month); and (d) ≥ 5 drinking episodes per month. BMI (kg/m2) was categorized in accordance with the WHO Asia-Pacific perspective as <18.5, <23, <25, and ≥25 through physical measurement. Use of medical care over the past year was evaluated separately as: (a) admission, and (b) ≥2 wks outpatient care. Level of daily activity was classified into 4 grades: sedentary, light, moderate and heavy/extreme. Bed rest for the past month, and absence from work for the past month indicated sick leave or bed rest at present or up to 1 month previous. Subjective health combined categories into 3, merging ‘very good’ and ‘good’ into ‘good’, ‘bad’ and ‘very bad’ to ‘bad’, and the rest into ‘fair’.

### Statistical analysis

We employed a complex sampling design analysis approach using stratified, cluster, and weighted variables, and all analyses were performed using statistical package SAS ver. 9.3 (SAS Institute Inc, Cary, NC, USA) with *p* < 0.05 considered to be statistically significant. Continuous variables were expressed as mean and standard deviation, and categorical variables as frequency count and percentage (%), and differences in sleep duration by characteristics were calculated using Rao-Scott chi-square test or ANOVA. The impact of mental health factors on sleep duration was assessed with a complex sampling design multinomial logistic regression analysis adjusting for covariates to obtain odds ratios (OR) and 95 % confidence intervals (CI).

### Ethics, consent and permissions

The interviewer was not given information about a participant before conducting the interview, and all participants gave written informed consent to participate. The protocol was approved by the Institutional Review Board of Jaseng Hospital of Korean Medicine in Seoul, Korea.

## Results

The average sleep duration in Koreans aged ≥19 yrs was 6.78 ± 1.41 h, and 6.82 ± 1.33 h in men and 6.75 ± 1.46 h in women. Sleep duration distribution was only 7.5 % (men 6.8 %; women 8.0 %) for ≥9 h, 41.9 % (men 41.6 %; women 42.1 %) for ≤6 h, and 50.7 % (men 51.6 %; women 50.0 %) for 7–8 h, showing the highest percentage.

In assessing the association between mental health and sleep duration in KNHANES participants, age, sex, socioeconomic (income, education, occupation), lifestyle-related (marital status, drinking, smoking), and health-related factors (subjective health, current chronic disease morbidity, BMI, use of medical care, level of daily activity, bed rest for the past month, absence from work for the past month) were included as covariates. All factors except smoking were shown to be associated with sleep duration (Table 1).

**Table 1:** Characteristics of Korean adults aged ≥19 years participating in KNHANES V (2010–2012) (*N* = 17,638)

	Sleep duration			
Variable	≤6 h	7–8 h	≥9 h	*p*-value^b^
	*n* = 7,384	*n* = 8,936	*n* = 1,318	
Age (mean ± SD)^a^	47.9 ± 16.0	44.1 ± 15.0	44.7 ± 18.2	<.001
Sex				
Male	3110 (41.6)	3861 (51.6)	511 (6.8)	0.001
Female	4274 (42.1)	5075 (50.0)	807 (8.0)	
Household income				
Low	1789 (42.2)	2054 (48.4)	397 (9.4)	<.001
Middle-low	1781 (40.3)	2270 (51.4)	364 (8.2)	
Mid-upper	1841 (41.6)	2282 (51.6)	301 (6.8)	
High	1889 (43.2)	2242 (51.3)	242 (5.5)	
Education				
≤8 years	2316 (50.6)	1816 (39.6)	449 (9.8)	<.001
9–11 years	833 (43.1)	953 (49.3)	148 (7.7)	
12–15 years	2209 (38.5)	3119 (54.3)	413 (7.2)	
≥16 years	2006 (37.6)	3028 (56.7)	304 (5.7)	
Employment				
Unemployed	3011 (41.9)	3499 (48.7)	680 (9.5)	<.001
Employed	4355 (41.9)	5418 (52.1)	634 (6.1)	
Marital status				
With spouse	5330 (40.6)	6863 (52.2)	949 (7.2)	<.001
Separated, divorced, bereaved	1227 (55.5)	833 (37.6)	153 (6.9)	
Single	541 (36.5)	800 (53.9)	142 (9.6)	
Body mass index				
<18.5	273 (34.7)	422 (53.7)	91 (11.6)	<.001
<23	2788 (39.6)	3681 (52.3)	567 (8.1)	
<25	1780 (43.3)	2064 (50.2)	269 (6.5)	
≥25	2511 (44.7)	2723 (48.5)	381 (6.8)	
Smoking status				
Never smokers	4520 (41.7)	5502 (50.8)	806 (7.4)	0.63
Exsmokers	1370 (42.5)	1610 (50.0)	242 (7.5)	
Current smokers	1491 (41.6)	1823 (50.9)	270 (7.5)	
Drinking				
Non alcohol consumption	2306 (45.5)	2339 (46.1)	424 (8.4)	<.001
<1 drinking episode per month	1369 (40.7)	1755 (52.2)	240 (7.1)	
<5 drinking episodes per month	2115 (38.7)	2964 (54.2)	386 (7.1)	
≥5 drinking episodes per month	1571 (42.9)	1824 (49.9)	264 (7.2)	
Level of daily activity				
Sedentary	789 (40.8)	872 (45.1)	272 (14.1)	<.001
Light	2924 (40.4)	3831 (52.9)	483 (6.7)	
Moderate	3032 (43.3)	3551 (50.7)	426 (6.1)	
Heavy/extreme	633 (43.9)	675 (46.8)	134 (9.3)	
Subjective health				
Good	2319 (40.0)	3113 (53.6)	372 (6.4)	<.001
Fair	3409 (40.9)	4328 (51.9)	599 (7.2)	
Bad	1641 (47.4)	1477 (42.7)	344 (9.9)	
Bed rest for the past month				
No	6684 (41.4)	8284 (51.3)	1166 (7.2)	<.001
Yes	684 (46.7)	633 (43.2)	148 (10.1)	
Absence from work for the past month				
No	4211 (41.0)	5427 (52.9)	631 (6.1)	<.001
Yes	277 (47.5)	256 (43.9)	50 (8.6)	

^a^There are no missing values for age

^b^ *P*-value calculated from ANOVA or Rao-Scott chi-square test for continuous and categorical variables

The results showed that mental health was related to sleep duration. Univariate analysis showed that subjects who reported mental health issues such as stress, depressive symptoms, thoughts of suicide, and mental health counseling were more likely to sleep ≤6 h or ≥9 h than the recommended 7–8 h. Related mental health items showed gender-specific patterns in multivariate analysis (fully adjusted model). Men with high stress levels reported short sleep duration (OR 1.43, 95 % CI 1.15–1.78), while women presented significant sleep duration differences in both low and high stress levels (with almost no stress as reference), and reported more normal sleep than subjects with longer sleep duration (≥9 h, low stress levels OR 0.71, 95 % CI 0.53–0.96), but shorter compared to subjects with normal sleep duration (≤6 h, high stress levels OR 1.71, 95 % CI 1.40–2.09; ≤6 h, low stress levels OR 1.23, 95 % CI 1.03–1.47). Depressive symptoms were not associated with sleep duration in men, and shorter sleep duration in women (OR 1.71, 95 % CI 1.40–2.09). Thoughts of suicide were related with short sleep duration in men (OR 1.39, 95 % CI 1.09–1.76), but showed no relationship in women. Mental health counseling displayed similar tendencies, with men receiving counseling reporting shorter or longer sleep duration (≤6 h OR 2.21, 95 % CI 1.18–4.14; ≥9 h OR 4.27, 95 % CI 1.57–11.58), but differences were not significant in women (Table 2).

**Table 2:** Association between sleep duration and mental health in Korean adults aged ≥19 years participating in KNHANES V

		Sleep durations				Comparison between ≤6 h and 7–8 h (Ref) of sleep			Comparison between ≥9 h and 7–8 h (Ref) of sleep		
	Factors^a^					OR (95 % CI)			OR (95 % CI)		
		≤6	7 ~ 8	≥9	*p*-value	Crude	Sociodemographic factors adjusted^b^	Fully adjusted (including chronic disease)	Crude	Sociodemographic factors adjusted^b^	Fully adjusted (including chronic disease)
Men		*n* = 3110	*n* = 3861	*n* = 511							
	Stress perception										
	High	833 (48.2)	797 (46.1)	100 (5.8)	<.0001	1.37 (1.14–1.65)	1.40 (1.14–1.72)	1.43 (1.15–1.78)	0.95 (0.67–1.33)	1.21 (0.82–1.78)	1.09 (0.73–1.64)
	Low	1768 (39.9)	2376 (53.6)	286 (6.5)		0.96 (0.81–1.13)	0.99 (0.82–1.20)	1.00 (0.82–1.22)	0.82 (0.62–1.09)	0.99 (0.73–1.35)	0.96 (0.70–1.31)
	Almost none	509 (38.5)	688 (52.0)	125 (9.5)							
	Depressive symptom morbidity										
	No	2806 (41.2)	3556 (52.2)	445 (6.5)	0.0011						
	Yes	304 (45.0)	305 (45.2)	66 (9.8)		1.20 (0.97–1.50)	1.18 (0.93–1.49)	1.26 (0.98–1.61)	1.96 (1.36–2.82)	1.38 (0.91–2.10)	1.37 (0.88–2.15)
	Thoughts of suicide										
	No	2754 (41.0)	3527 (52.5)	443 (6.6)	0.0006						
	Yes	356 (47.0)	334 (44.1)	68 (9.0)		1.38 (1.13–1.69)	1.34 (1.07–1.67)	1.39 (1.09–1.76)	1.76 (1.23–2.51)	1.32 (0.90–1.93)	1.42 (0.94–2.15)
	Psychiatric counseling										
	No	3059 (41.5)	3818 (51.8)	499 (6.8)	<.0001						
	Yes	51 (48.1)	43 (40.6)	12 (11.3)		1.59 (0.95–2.68)	1.75 (1.00–3.07)	2.21 (1.18–4.14)	4.68 (2.17–10.12)	3.44 (1.39–8.52)	4.27 (1.57–11.58)
Women	*n* = 4274	*n* = 5075	*n* = 807								
	Stress perception										
	High	1404 (48.0)	1294 (44.2)	229 (7.8)	<.0001	1.29 (1.10–1.52)	1.78 (1.46–2.16)	1.71 (1.40–2.09)	0.83 (0.62–1.11)	0.67 (0.49–0.93)	0.74 (0.52–1.04)
	Low	2239 (39.1)	3066 (53.5)	423 (7.4)		0.90 (0.77–1.04)	1.25 (1.05–1.49)	1.23 (1.03–1.47)	0.67 (0.52–0.87)	0.68 (0.51–0.90)	0.71 (0.53–0.96)
	Almost none	630 (42.1)	711 (47.6)	154 (10.3)							
	Depressive symptom morbidity										
	No	3476 (41.1)	4343 (51.3)	648 (7.7)	<.0001						
	Yes	797 (47.3)	731 (43.4)	158 (9.4)		1.41 (1.23–1.60)	1.21 (1.05–1.40)	1.71 (1.40–2.09)	1.55 (1.22–1.96)	1.19 (0.92–1.55)	0.74 (0.52–1.04)
	Thoughts of suicide										
	No	3405 (40.9)	4281 (51.4)	647 (7.8)	<.0001						
	Yes	869 (47.8)	790 (43.5)	159 (8.8)		1.39 (1.22–1.58)	1.12 (0.97–1.30)	1.13 (0.98–1.30)	1.37 (1.07–1.76)	1.00 (0.76–1.32)	1.00 (0.76–1.31)
	Psychiatric counseling										
	No	4135 (42.0)	4947 (50.2)	775 (7.9)	0.1502						
	Yes	139 (46.8)	126 (42.4)	32 (10.8)		1.12 (0.83–1.51)	1.10 (0.81–1.51)	1.13 (0.83–1.55)	1.56 (0.99–2.46)	1.14 (0.68–1.90)	1.14 (0.69–1.87)

Multinomial logistic regression analysis

^a^Calculated using Rao-scott chi-square test

^b^Adjusted for age, sex, household income, education, marital status, employment, BMI, smoking, alcohol consumption, level of daily activity, subjective health, bed rest for the past month and absence from work for the past month

Fully adjusted models of mental health showed that any chronic disease morbidity was correlated with ≤6 h sleep (OR 1.10, 95 % CI 1.00–1.21), and osteoarthritis (OR 1.23, 95 % CI 1.07–1.41) and cancer with short sleep duration (OR 1.38, 95 % CI 1.08–1.77), while diabetes was associated with normal sleep (compared to short sleep duration) (OR 0.79, 95 % CI 0.69–0.92), and dyslipidemia with normal sleep duration (compared to long sleep duration) (OR 0.71, 95 % CI 0.54–0.94) (Table 3).

**Table 3:** Association between sleep duration and chronic diseases in Korean adults aged ≥19 years participating in KNHANES V (2010–2012)

		Comparison between ≤6 h and 7–8 h (Ref) of sleep			Comparison between ≥9 h and 7–8 h (Ref) of sleep		
		N	Crude	Fully adjusted^a^	N	Crude	Fully adjusted^a^
Factors			OR (95 % CI)	OR (95 % CI)		OR (95 % CI)	OR (95 % CI)
Hypertension	No	12417 (5353)			8062 (998)		
	Yes	3774 (1977)	1.39 (1.27–1.52)	0.95 (0.86–1.06)	2106 (309)	1.05 (0.88–1.26)	0.86 (0.7–1.06)
Dyslipidemia	No	14369 (6359)			9218 (1208)		
	Yes	1809 (958)	1.41 (1.24–1.6)	1.1 (0.96–1.26)	954 (103)	0.83 (0.64–1.08)	0.71 (0.54–0.94)
Stroke	No	15953 (7199)			10042 (1288)		
	Yes	313 (160)	1.23 (0.94–1.62)	0.86 (0.65–1.15)	176 (23)	0.93 (0.56–1.55)	0.62 (0.37–1.05)
Cardiovascular disease	No	15769 (7090)			9953 (1274)		
	Yes	464 (246)	1.53 (1.23–1.91)	1.12 (0.89–1.41)	253 (35)	0.95 (0.61–1.48)	0.73 (0.45–1.17)
Osteoarthritis	No	13860 (5986)			8987 (1113)		
	Yes	1911 (1101)	1.8 (1.6–2.03)	1.23 (1.07–1.41)	970 (160)	1.34 (1.07–1.68)	0.97 (0.75–1.25)
Rheumatoid arthritis	No	15875 (7143)			10009 (1277)		
	Yes	332 (184)	1.46 (1.1–1.95)	1.1 (0.81–1.48)	176 (28)	1.47 (0.88–2.48)	1.2 (0.7–2.06)
Diabetes	No	14887 (6654)			9408 (1175)		
	Yes	1360 (695)	1.13 (0.99–1.3)	0.79 (0.69–0.92)	802 (137)	1.31 (1.03–1.66)	1 (0.77–1.32)
Atopic dermatitis	No	15574 (7058)			9768 (1252)		
	Yes	342 (163)	1.12 (0.87–1.46)	1.26 (0.95–1.66)	220 (41)	1.7 (1.08–2.67)	1.49 (0.89–2.52)
Pulmonary tuberculosis	No	15351 (6886)			9716 (1251)		
	Yes	794 (404)	1.31 (1.07–1.6)	1.21 (0.98–1.5)	445 (55)	1.14 (0.8–1.63)	1.06 (0.72–1.57)
Asthma	No	15570 (6986)			9824 (1240)		
	Yes	522 (282)	1.34 (1.08–1.67)	1.16 (0.93–1.46)	291 (51)	1.67 (1.15–2.44)	1.27 (0.84–1.91)
Thyroid-related disorders	No	15604 (7029)			9843 (1268)		
	Yes	659 (328)	1.27 (1.05–1.54)	1.16 (0.95–1.41)	376 (45)	1.04 (0.69–1.59)	0.95 (0.62–1.45)
Cancer	No	15778 (7097)			9954 (1273)		
	Yes	504 (269)	1.61 (1.27–2.04)	1.38 (1.08–1.77)	275 (40)	1.4 (0.9–2.17)	1.08 (0.67–1.72)
Hepatitis	No	15966 (7227)			10039 (1300)		
	Yes	304 (136)	0.85 (0.63–1.14)	0.83 (0.61–1.12)	181 (13)	0.53 (0.26–1.07)	0.6 (0.3–1.21)
Any experience of chronic disease	No	8077(3262)			5477(662)		
	Yes	6672(3358)	1.40 (1.30–1.52)	1.10 (1.00–1.21)	3851(537)	1.11 (0.96–1.29)	0.94 (0.77–1.15)

Multinomial logistic regression analysis

^a^Adjusted for age, sex, household income, education, marital status, employment, BMI, smoking, alcohol consumption, level of daily activity, subjective health, bed rest for the past month, absence from work for the past month, stress, depressive symptoms, thoughts of suicide

## Discussion

The results show that stress, experience of depressive symptoms, thoughts of suicide, and psychiatric counseling were all associated with short or long sleep duration. Mental health factors and sleep duration associations revealed gender-specific patterns, with thoughts of suicide and psychiatric counseling more related in men, and stress and depressive symptoms in women. It has been generally acknowledged that one of the reasons women are more susceptible to depression than men is the direct influence of follicular hormones [15, 16]. Other explanations include that the HPA axis which regulates stress is more dysfunctional in women [17], and that follicular hormones affect HPA regulation [18]. Most women experience premenstrual symptoms over their lifetime, and 1/5 report serious symptoms including depression [19]. Also, erratic cyclic variation in estrogen and progestogen levels occurs in perimenopausal and postmenopausal periods, rendering menopausal women susceptible to major depression, and this tendency is more pronounced in women with a history of depressive episodes [20]. Women also differ in response and adaptation to stress, and adolescent girls have been shown to focus more internally on stressful emotions and mental distress [21].

Meanwhile, men tend to be more impulsive and prone to high-risk behavior than women [22]. Additionally, men are more prone to antisocial personality disorders, behavioral disorders, ADHD, and intermittent explosive disorders [23–27].

Associations were found between sleeping ≤6 h and higher levels of stress in women, and in ≥9 h sleep and lower stress levels. Although the causal relationship regarding short sleep and stress has not been established, recent studies point toward a bi-directional association [6, 28, 29].

Magee et al. purported that short sleep duration could induce more fatigue or mood and cognition disturbances, resulting in poor self-rated health (SRH) or quality of life (QOL) [29]. Subjects with poorer health notably had more sleep disturbance and short sleep duration, and Kessler et al. reported that significantly more subjects sleeping 7–8 h a day had low scores (<16) in the Kessler Psychological Distress Scale than those with short or long sleep duration [26].

Thoughts of suicide were associated with both short and long sleep duration in this study, whereas suicidal intentions and increased risk of suicide attempt have been shown to be related with short sleep in previous literature [7–10], and a 2001 review concluded that sleep disturbance was more frequent in psychiatric patients displaying suicidal behavior [30].

Although the underlying mechanism linking short sleep duration and suicide attempts is not yet known, insufficient sleep is known to have negative effects on cognitive function, resulting in impaired judgment and increased compulsion, exhaustion, and despair [8, 31], placing patients under greater risk of impulsive behavior [11].

This study found that while women sleeping ≤6 h experienced more depressive symptoms than those sleeping 7–8 h, men did not show association between depressive symptoms and sleep duration.,

Gangwisch et al. purported that depression was associated with both long and short sleep duration [32]. Martin-Merino et al. suggested that sleep-related problems were a strong indicator of depression, and deprivation of sleep may alter mood and individual wellbeing [33]. Patel et al. reported that longer sleep was associated with higher risk of depression, use of antidepressants and benzodiazepine [34], which may be due, in part, to the sedative effect of psychotropic medication [35].

Psychiatric counseling showed significant correlation with short or long sleep duration in men. Men typically show weaker restraint over impulse, and higher prevalence of psychological disorder presenting overt or impulsive behavior [23–27], which may be why psychiatric counseling was also more significant in men. However, the total prevalence of psychiatric counseling was much lower than that of depressive symptoms, thoughts of suicide, and stress, indicating that many patients with mental health problems were not receiving appropriate counseling.

Many studies have addressed the relationship between chronic disease and mental health: it has been reported that 50 % of cancer patients suffer from various mental health issues, depression patients are 2 times more likely to have a heart attack than non-depressive individuals [36], and type II diabetes mellitus patients have a two-fold higher risk of depression than non-diabetics [37]. Also, chronic disease has been shown to be a major factor for low HRQoL through EQ-5D studies in chronic disease patients [38]. While some report an inverse association between chronic disease and mental health, some state that mental health remains stable and unaffected by chronic disease [39].

Some previous studies have reported relationships between stress-prone personalities and cardiovascular risk factors [40, 41]. Stress is associated with total cholesterol, low-density lipoprotein and triglycerides [42], and modern stress-ridden lifestyles may seriously impact metabolism and may bring on pathological changes [43]. Prolonged changes in lipid metabolism from chronic stress may lead to cardiovascular disease [44].

We adjusted for mental health to adjust for the potential relationship between chronic disease and mental health. Participants with any chronic disease, and participants with osteoarthritis, diabetes, dyslipidemia, or cancer out of the various chronic diseases were shown to have significant relations with sleep duration, and osteoarthritis and cancer patients showed higher prevalence in short sleep duration. Diabetes was associated with normal sleep (compared to short sleep duration), and dyslipidia with normal sleep duration (compared to long sleep duration).

Numerous studies have been conducted on the association between chronic disease and sleep duration. A large-scale cohort on 71,617 women aged 45 to 65 conducted by Ayas et al. concluded that both long and short sleep duration was associated with cardiovascular disease [14]. Given the sex and age group, it is highly probable that many, if not most, participants were menopausal, while this study included both male and female adults, and did not find a significant relationship between cardiovascular disease and sleep duration.

The results of a cross-sectional study investigating chronic disease and sleep duration in the Brazilian population are consistent with this study in reporting the association between depression and anxiety with sleep duration, but it failed to adjust for other mental health variables [13].

Dyslipidemia and diabetes were shown to be associated with normal sleep duration, indicating less association with abnormal sleep duration. This is contrary to previous studies which purport positive correlations between short sleep duration and dyslipidemia [45] and diabetes [46], while Kaneita reported in a 2008 study that subjects with short or long sleep duration had higher serum triglyceride and lower high-density lipoprotein cholesterol levels than those with appropriate sleep [47]. Other studies have put forth that longer sleep duration may increase diabetes incidence, or that longer sleep duration itself may be an early symptom of diabetes [48].

Osteoarthritis was shown to be related to short sleep duration as in a previous Brazilian study where prevalence of shorter or longer sleep duration was higher in osteoarthritic, rheumatic, osteoporotic, and arthritic subjects, and the reason was suggested to be disease-related pain and consequent disruption of sleep patterns [13].

Our study also revealed that cancer was associated with short sleep, contrary to reports from previous studies. One meta-analysis concluded that short sleep duration was unrelated to cancer, and though longer sleep duration displayed a positive relationship with colorectal cancer occurrence, it was found to reduce risk in hormone-associated cancers [49]. The association between long sleep duration and cancer incidence may vary by course, and more cancer patients of <5 yrs duration presented with depressive symptoms compared to those of ≥5 yrs [50]. Mental health is highly relevant in cancer progress as it entails high mortality risks, and should be considered in future studies.

Some major strengths of this study are that the population is a nationally representative sample of Koreans, and the health examinations and surveys were conducted by prior trained surveyors. Also, many factors that may confound sleep duration, mental health, and chronic disease (age, sex, income, education, employment, marital state, smoking, drinking habits, subjective health, BMI, use of medical care, level of daily activity, bed rest for the past month, absence from work for the past month) were included for adjustment. We would also like to draw attention to the fact that we were able to determine gender-specific patterns in relation to sleep duration by considering various aspects of mental health, and that we gave consideration to chronic disease in assessing the relationship between mental health with sleep duration and vice versa, including mental health factors as confounding variables in associations between sleep and chronic disease.

Meanwhile, the biggest limitation of our study is due to its cross-sectional design. Though causal relationships cannot be deduced, these results may be used as basic data offering clues to how chronic disease, mental health and sleep duration is associated and underlying physiological and biochemical mechanisms. Other limitations include that while national-level surveys hold considerable power, the data entails certain limitations as questions and diagnoses have to be kept relatively simple, the validity and reliability of data could be questionable as data collection relied on self-report, and quality of sleep or history of sleep-related disorders were not investigated, thus restricting full evaluation of sleep disturbance problems. For example, only 4 items were related to mental health, and criteria for stress perception is somewhat ambiguous as it was assessed through subjective self-evaluation of high, low, or almost no stress as opposed to objective criteria or professional opinion. In addition, there was overlapping between the constructs of depressive symptoms, suicidal thoughts, psychiatric counseling, and stress. For instance, suicidal thoughts and sleep disturbance may be symptoms of depression or stress.

Still, the authors performed secondary analyses by group (only depressive symptoms, depressive symptoms with psychiatric counseling, depressive symptoms with suicidal thoughts, and depressive symptoms with psychiatric counseling and suicidal thoughts) hypothesizing that such additional analyses would reflect severity of depressive symptoms and stronger association with sleep duration. However, statistically significant difference shown in crude analysis was not maintained in adjusted analyses. This may be due to decreased sample size or as the possible pathways between such constructs as counseling and sleep disturbance are likely to be bi-directional (i.e. disturbed sleep is likely to be associated with increased chances of seeking psychiatric counseling, but psychiatric counseling should be associated with a subsequently decreased level of disturbed sleep) (see Additional file 1).

While the present study interpreted gender-specific patterns to be due to biological differences such as hormones, future studies may give more consideration to social gender roles and psychosocial differences between men and women in study design and interpretation of mental health and sleep pattern findings. For example, men may be more reluctant to seek psychiatric counseling and thus men seeking treatment may be more adversely affected than women. Further multidimensional studies should also be conducted with thorough consideration of construct and measures and evaluate the mediation effect of mental health in the association between chronic disease and sleep.

## Conclusions

In conclusion, poor mental health was associated with short or long sleep duration, showing significance in contemplation of suicide, psychiatric counseling and stress in men, and stress and depressive symptoms in women. After adjusting for covariates including mental health factors, chronic disease morbidity, osteoarthritis, and cancer showed relationships with short or long sleep duration. Diabetes was associated with normal sleep (compared to short sleep duration), and dyslipidia with normal sleep duration (compared to long sleep duration). Further prospective studies are required to identify the pathways and causal relations by which chronic disease is associated with sleep duration.

## Availability of data and materials

The KNHANES data is third-party data not owned by the authors. The KNHANES annual reports, user manuals, and raw data are accessible at the ‘Korea National Health and Nutrition Examination Survey’ website (https://knhanes.cdc.go.kr/knhanes/index.do) through email request.

## Additional file

Additional file 1:
**Association between sleep duration and mental health by mental health group in Korean adults aged ≥19 years participating in KNHANES V.** (DOCX 50 kb)

## Abbreviations

KNHANES: Korean National Health and Nutrition Examination Survey
BMI: body mass index
WHO: World Health Organization
ANOVA: analysis of variance
OR: odds ratio
CI: confidence interval
SRH: self-rated health
QOL: quality of life
