# Supplementary material for: The association between mental health, chronic disease and sleep duration in Koreans: a cross-sectional study
Source: BMC Public Health. 2015 Dec 1;15:1200. doi: 10.1186/s12889-015-2542-3 (PMC4665819; doi:10.1186/s12889-015-2542-3)
Supplement: Additional file 1: — Association between sleep duration and mental health by mental health group in Korean adults aged ≥19 years participating in KNHANES V. (DOCX 50 kb) [file 12889_2015_2542_MOESM1_ESM.docx]

Additional table 1. Association between sleep duration and mental health by mental health group in Korean adults aged ≥19 years participating in KNHANES V

|  |  | Sleep durations | | | |  | Comparison between  ≤6 hrs and 7-8 hrs (Ref) of sleep | |  | Comparison between  ≥9 hrs and 7-8 hrs (Ref) of sleep | |
| --- | --- | --- | --- | --- | --- | --- | --- | --- | --- | --- | --- |
|  | Factors^a^ |  |  |  |  |  | OR (95% CI) | |  | OR (95% CI) | |
|  |  | ≤6 | 7~8 | ≥9 | p-value |  | Sociodemographic factors adjusted^b^ | Fully adjusted(including chronic disease) |  | Sociodemographic factors adjusted^b^ | Fully adjusted(including chronic disease) |
| Men | | n=3110 | n=3861 | n=511 |  |  |  |  |  |  |  |
|  | Only depressive symptoms |  |  |  |  |  |  |  |  |  |  |
|  | No | 2983 (41.7) | 3698 (51.7) | 474 (6.6) | 0.1121 |  |  |  |  |  |  |
|  | Yes | 127 (38.8) | 163 (49.9) | 37 (11.3) |  |  | 1.00 (0.74-1.35) | 1.05 (0.76-1.44) |  | 1.29 (0.76-2.20) | 1.18 (0.68-2.06) |
|  | Depressive symptoms with psychiatric counseling |  |  |  |  |  |  |  |  |  |  |
|  | No | 3100 (41.6) | 3850 (51.6) | 510 (6.8) | 0.731 |  |  |  |  |  |  |
|  | Yes | 10 (45.5) | 11 (50) | 1 (4.6) |  |  | 1.44 (0.50-4.14) | 1.82 (0.58-5.75) |  | 1.65 (0.23-11.67) | 2.14 (0.30-15.09) |
|  | Depressive symptoms with suicidal thoughts |  |  |  |  |  |  |  |  |  |  |
|  | No | 2958 (41.2) | 3742 (52.1) | 487 (6.8) | 0.0069 |  |  |  |  |  |  |
|  | Yes | 152 (51.5) | 119 (40.3) | 24 (8.1) |  |  | 1.32 (0.94-1.85) | 1.40 (0.97-2.01) |  | 1.20 (0.67-2.14) | 1.28 (0.68-2.40) |
|  | Depressive symptoms with psychiatric counseling and suicidal thoughts |  |  |  |  |  |  |  |  |  |  |
|  | No | 3095 (41.5) | 3849 (51.7) | 507 (6.8) | 0.0237 |  |  |  |  |  |  |
|  | Yes | 15 (48.4) | 12 (38.7) | 4 (12.9) |  |  | 1.65 (0.57-4.76) | 1.96 (0.57-6.76) |  | 3.29 (0.69-15.72) | 3.6 (0.58-22.56) |
| Women | | n=4274 | n=5075 | n=807 |  |  |  |  |  |  |  |
|  | Only depressive symptoms |  |  |  |  |  |  |  |  |  |  |
|  | No | 3956 (41.9) | 4744 (50.3) | 741 (7.9) | 0.0351 |  |  |  |  |  |  |
|  | Yes | 318 (44.5) | 330 (46.2) | 66 (9.2) |  |  | 1.17 (0.95-1.45) | 1.15 (0.91-1.45) |  | 1.22 (0.85-1.75) | 1.24 (0.84-1.81) |
|  | Depressive symptoms with psychiatric counseling |  |  |  |  |  |  |  |  |  |  |
|  | No | 4252 (42.1) | 5053 (50) | 806 (8) | 0.6832 |  |  |  |  |  |  |
|  | Yes | 22 (50) | 21 (47.7) | 1 (2.3) |  |  | 0.76 (0.39-1.50) | 0.69 (0.32-1.47) |  | 0.51 (0.06-4.50) | 0.56 (0.06-5.20) |
|  | Depressive symptoms with suicidal thoughts |  |  |  |  |  |  |  |  |  |  |
|  | No | 3876 (41.5) | 4742 (50.7) | 732 (7.8) | <.0001 |  |  |  |  |  |  |
|  | Yes | 398 (49.4) | 332 (41.2) | 75 (9.3) |  |  | 1.22 (0.99-1.49) | 1.23 (0.99-1.53) |  | 1.19 (0.84-1.68) | 1.27 (0.87-1.86) |
|  | Depressive symptoms with psychiatric counseling and suicidal thoughts |  |  |  |  |  |  |  |  |  |  |
|  | No | 4215 (42) | 5028 (50.1) | 791 (7.9) | 0.0883 |  |  |  |  |  |  |
|  | Yes | 59 (48.8) | 46 (38) | 16 (13.2) |  |  | 1.31 (0.80-2.16) | 1.37 (0.80-2.36) |  | 1.17 (0.60-2.31) | 1.32 (0.63-2.75) |

Multinomial logistic regression analysis

^a^ Calculated using Rao-scott chi-square test

^b^ Adjusted for age, sex, household income, education, marital status, employment, BMI, smoking, alcohol consumption, level of daily activity, subjective health, bed rest for the past month and absence from work for the past month
